# What are the clinical practice experiences of specialist and advanced paramedics working in emergency department roles? A qualitative study

**DOI:** 10.29045/14784726.2019.12.4.3.1

**Published:** 2019-12-01

**Authors:** Alan Clarke

**Affiliations:** University of Wolverhampton

**Keywords:** advanced clinical practice, advanced clinical practitioner, emergency care practitioner, emergency department, paramedic, prescribing

## Abstract

**Aim:**

Little is known about paramedics who have left the ambulance service to work in emergency departments (EDs). This study sought to explore the lived experiences of paramedics working in specialist/advanced ED roles, focusing on role transition, influences on effective clinical practice and perceptions of role optimisation. A secondary aim of the study was to make recommendations on the future development of specialist/advanced ED roles for paramedics.

**Methods:**

This was a qualitative study utilising descriptive phenomenology to collect and describe the lived experiences of participants via semi-structured interviews. The final sample comprised three emergency care practitioners (ECPs), three student ECPs and two advanced clinical practitioners (ACPs), all Health and Care Professions Council registered paramedics. Interview data were transcribed verbatim and analysed using inductive thematic analysis.

**Results:**

- Transition to the ED involves significant adjustment to a new clinical environment, responsibilities and decision making.
- Pre-hospital physical assessment and history taking skills, and experience of autonomous working are pertinent enablers to effective practice within the ED.
- Difficulties in accessing medication in the ED emerged as a significant barrier to daily practice that could affect the patient experience and influence perceptions of sub-optimal working.
- Misconceptions by ED staff regarding paramedic competencies could lead to role confusion and make inter-professional working difficult.
- Opportunities exist for future role expansion into areas such as resus, majors and paediatrics within the ED environment.

**Conclusions:**

While role transition to the ED represents a turbulent period for paramedics, elements of pre-hospital paramedic practice transfer directly into ED roles and contribute to effective practice. Participants found that they were accepted and supported to work in the ED setting and spoke positively of future role expansion. A lack of access to medicines presents a significant barrier to current clinical practice and a disparity in practice between paramedics and their nursing counterparts. The change in legislation to allow independent prescribing for advanced paramedics will address some of these issues, but interim improvements are required to extend existing arrangements to paramedics, improving the quality and safety of care they provide and ultimately the patient experience.

## Introduction

Since the inception of the paramedic role, the clinical practice of paramedics in the United Kingdom has undergone a paradigm shift as the profession has become integrated into the wider emergency care arena, with the development of specialist and advanced paramedic roles (Gallagher et al., 2016). An increasing demand for emergency care services, an ageing population with complex health needs, the devolution of skills from doctors to other professions, as well as the political resolve to reform existing models of care have been pivotal in this change in scope of practice for paramedics (Mason et al., 2012; National Audit Office, 2011; NHS England, 2013; Tavares, Bowles, & Donelon, 2016). The emergency care practitioner (ECP) and advanced clinical practitioner (ACP) are two examples of roles that can be taken up by paramedics (and other health professions) designed to better manage the complete clinical care of patients presenting to areas such as emergency departments (EDs), minor injury units and out-of-hours services (Health Education England, 2017; Hill, McMeekin, & Price, 2014; Mason, O’Keeffe, Coleman, Edlin, & Nicholl, 2007; Mason et al., 2012; NHS England, 2013). In addition to the ECP being deployed by ambulance services to better manage low acuity calls, they are now being utilised in other areas such as EDs and urgent care centres, allowing paramedics to move out of ambulance services and practise in a variety of alternative areas and roles (College of Paramedics, 2015), the profession now reflecting the diversity of paramedic practice found in other countries (Perchie, 2003).

Despite a variety of UK studies that have examined the pre-hospital efficacy of ECPs, less is known about alternative areas of practice. For example, ECPs are proven to be effective at treating and discharging patients without referral to other healthcare professionals in the pre-hospital context, but less effective in this respect when working in out-of-hours or urgent care services (Mason et al., 2012); it is not clear though what influences there are on practice that may contribute to this. Furthermore, findings from overseas studies and from studies of other UK ED roles, such as the emergency nurse practitioner or advanced nurse practitioner, cannot be generalised to paramedics due to differences in UK service delivery, professional scope of practice and education (Bryson, 2016; Fisher, 2006; Griffin & Melby, 2006; Lloyd-Rees, 2016; Norris & Melby, 2006; Perchie, 2003; Tye & Ross, 2000).

As such, there is a need for research that explores the experiences of UK specialist/advanced paramedics, practising in alternative settings, to inform future role and practice development. The aim of this study was to explore the lived experiences of paramedics who have made the transition from the ambulance service to specialist/advanced ED roles in the United Kingdom, and to explore how working in this new clinical environment influenced their clinical practice.

## Methods

The consolidated criteria for reporting qualitative research (COREQ), which are an EQUATOR Network approved reporting guideline for interviews and focus groups, have been used for the reporting of this original research study (Tong, Sainsbury, & Craig, 2007).

### Study design

A qualitative, descriptive phenomenological approach was chosen for this study. This focuses on the everyday experiences of a phenomenon in order to gain understanding of its essence via the description and analysis of the lived experiences of participants. Such a methodology was appropriate considering the nascent knowledge of paramedic specialist/advanced practice outside of the pre-hospital context (Converse, 2012; Punch, 2006).

### Target population and sampling

Two target populations were defined: paramedics working in the EDs within the West Midlands region and student emergency practitioners enrolled at a West Midlands university, who had worked in an ED during their emergency practitioner course. Participants were recruited via advertisement within EDs, direct invitation and face-to-face briefings. Purposive and convenience sampling was used to select the final sample consisting of three ECPs, three student ECPs and two ACPs, all Health and Care Professions Council registered paramedics. The ECP and ACP participants were not known to the researcher prior to the study; however, the student ECPs were recruited from the researcher’s faculty and had been taught by the researcher during their course. The inclusion and exclusion criteria employed during sampling is shown in Table 1. Demographic data relating to the study participants and participant code are shown in Table 2.

**Table 1.** Inclusion/exclusion criteria for the study sample.

Inclusion criteria	Exclusion criteria
HCPC registered paramedics that are either: A qualified emergency practitioner/ACP, practising in one of the target EDs within the West Midlands region. Enrolled as a student emergency practitioner and completed or currently undertaking an ED placement.	Any other health professional working as or studying to be an emergency practitioner or ACP, i.e. not a registered HCPC paramedic. Paramedics with a dual registration, giving them a potentially different scope of practice. Paramedics enrolled as a student emergency practitioner but yet to undertake a placement within an ED and who would have no experience of working in an ED.

ACP = advanced clinical practitioner; ED = emergency department; HCPC = Health and Care Professions Council.

**Table 2.** Profile of the study participants.

Participant code	Current role	Approximate length of time in ED (months)	Gender
P1	ECP	12	M
P2	Student ECP	2	F
P3	ECP	14	F
P4	ECP	10	M
P5	ACP	24	M
P6	Student ECP	2	M
P7	Student ECP	2	M
P8	ACP	24	F

ACP = advanced clinical practitioner; ECP = emergency care practitioner; ED = emergency department; F = female; M = male.

### Data collection

Data were collected via semi-structured interviews held either at the participant’s workplace or, for student emergency practitioners, their university campus to ensure a familiar environment. A semi-structured interview guide allowed flexibility in the questioning and exploration of salient responses in more detail as they emerged (Choo, Garro, Ranney, Meisel, & Morrow Guthrie, 2015; Ritchie, Lewis, McNaughton-Nicholls, & Ormston, 2014). All interviews were held in private, audio recorded and transcribed verbatim ensuring participant anonymity and organisational confidentiality, participant identity being replaced by Participant 1, 2 and so on, on interview transcripts and in the final presentation of the study findings.

### Data analysis

An inductive thematic approach to data analysis was employed using the data analysis spiral described by Creswell and Poth (2018); this provides a comprehensive framework and reflects the iterative nature of qualitative data analysis (Kodish & Gittelsohn, 2011). Firstly, bracketing allowed the researcher to acknowledge their own experiences and beliefs relating to the phenomena and their potential to influence the interpretation of study data. Following this, each audio recording was transcribed in a close to verbatim style and stored electronically. The decision was taken not to return transcripts to participants for member checking as perceptions and recall of phenomena are subject to change over time, leading to the researcher then trying to assimilate potentially disconfirming data (Birt, Scott, Cavers, Campbell, & Walter, 2016). Interview transcripts were read several times in order to gain familiarity with the content and immersion in the narrative. Pertinent statements were highlighted and memos added to the transcripts consisting of initial concepts and impressions, facilitating the formation of tentative codes (Creswell & Poth, 2018). The coding process was reinforced by employing a modified version of the double coding strategy suggested by Ranney et al. (2015); initial coding was reviewed by the research supervisor for accuracy and to detect potential bias. Codes and memos were subsequently compared and those with similar meaning grouped together into themes and sub-themes. The study findings were presented as a descriptive analysis representing the lived experiences of the participants in a narrative manner, achieved by adding the anonymised verbatim responses of the participants to the relevant sub theme.

## Results

Three broad themes emerged from the thematic data analysis spiral related to the areas explored during the qualitative interviews: (a) role transition; (b) influences on clinical practice; and (c) role optimisation and development.

### Role transition

In this first theme, opportunities for career development within the ambulance service were criticised and concerns over keeping up with the physical demands of ambulance work were voiced by many participants in relation to their decision to leave. For example, Participant 2 said:

I didn’t feel like there was much progression [in the ambulance service], apart from like mentoring and management. In terms of clinical areas, I didn’t feel like there was much progression at all for me to go into. (Participant 2)

Participant 5 commented on the physical demands:

The physical barriers of working in the ambulance service for 20-odd years and continuing to do so for another 20 years before retirement was motivation as well, so as [with] most ambulance people bad backs, bad knees, bad shoulders … (Participant 5)

Arriving in the ED exposed participants to the realities of working in this new setting, and the contrasts with their previous pre-hospital roles became apparent, such as managing multiple ED patients, as noted by Participant 4:

I find it [ED] a lot more manic, a lot busier than the ambulance service, this is like constant (…) there’ll be one patient in the X-ray and you’ll be doing two, three patients at a time maybe. (Participant 4)

When working in the ED, many participants described a steep learning curve while adjusting to new ways of working and putting new theory into practice, as can be seen in the comments of Participants 1 and 4:

You’re on the receiving end of what the paramedics bring in now; you need to see that injury, examine it and then determine what you need to do with it, whether you need to X-ray it, CT scan. (Participant 1)

Looking at X-rays, when you come to do it for real, you realise how much, probably little knowledge you do have, (…) it’s been a bit of a steep learning curve over the last year to get to where I am now. (Participant 4)

This transition process from pre-hospital to hospital also caused a sense of role regression for one participant, due to a perceived lack of knowledge:

I was very comfortable on the road, an expert in my field, and then you come into the hospital and I turned into that person who knew nothing. When a consultant asked me a question my mind just went blank through sheer panic. Being in the resus room I found really nerve racking because I didn’t like it. I just wanted to be anywhere else other than in a hospital. (Participant 8)

### Influences on clinical practice

In this second theme, there was agreement among the participants that certain skills and attributes from paramedic practice transferred directly into their new roles. For example, Participant 6 observed that:

History taking and [physical] assessment skills are always very similar regardless of where you are working. (Participant 6)

Participant 7 noted that previously acquired skills and knowledge in triage were still relevant:

We’re very good at triage and gathering that information in quickly, diagnostically examining it and saying this is what we think. (Participant 7)

All participants spoke of the difficulties they encountered when trying to obtain medications for their patients, most perceiving this as the most significant barrier to ED practice and voicing frustration at having to hand this over to colleagues. For example, Participant 3 said, when comparing pre-hospital practice to hospital practice:

The biggest [barrier] is medicines management, from being able to cannulate and give an IV medication; I can’t give anything now. (Participant 3)

Participant 5 noted that normal hospital procedures for administering medicines to patients such as patient group directions (PGDs) often excluded paramedics:

All of the PGDs within the department are written for registered nurses and they don’t mention paramedics specifically. There’s nothing within the hospital’s medicines administration policy at the moment regarding paramedics using JRCALC drugs within the hospital. (Participant 5)

Participants also spoke of how this affected their practice and the patient experience through delays occurring. For example, Participant 1 said:

It does happen a couple of times a day [medication access delays], two or three times a day then you’re looking at 15 to 45 minutes [delay] where you could be seeing someone else. (Participant 1)

There was agreement among the majority of participants over the benefits of being able to access medicines via PGD or for them being able to prescribe medicines, which was summarily noted by Participant 5:

Being able to prescribe it [medicines] and ask the nurses to give it immediately would be a huge benefit and obviously the more acute the patient presentation the more important it is we give these medicines and things in a timely manner. (Participant 5)

Furthermore, in this theme participants also found that their prior experience and competency as paramedics may not be recognised. One example of this is described by Participant 8, speaking about blood sugar analyses:

When I first came to the hospital I wasn’t allowed to do a blood sugar and I wasn’t allowed to give paracetamol because they weren’t quite sure what to do with me as a paramedic (…) all those things that actually I would have done every day [as a pre-hospital paramedic]. (Participant 8)

Conversely, Participant 5 found that ED colleagues could at times overestimate his previous experience and competence in regard to ED working, noting:

A lot of people have said to me ‘oh you don’t need to work in resus because you’ll be really good at that because of being a paramedic’ (…) but actually when it comes to the really ill patients [in ED] you have to be able to interact with people who are more experienced, more qualified on a level playing field, which as a paramedic I struggle with. (Participant 5)

### Role optimisation and development

In this third theme there were mixed opinions among the participants over whether they were able to practise to their full potential in their new ED roles. For one participant, the support from ED staff and a culture that encouraged achievement contributed to their perception of optimal working:

Yeah, absolutely and beyond that, there’s always somebody that’s willing to teach you and you will be pushed to achieve your full potential (…) there is a kind of buoyancy here to get you doing things and get you involved. (Participant 8)

For others, such as Participant 3, delayed access to medicines emerged as the primary reason for feeling that they could not perform their roles effectively:

Not to my full potential, not even in the slightest. The fact that we have to delay treatment to give anaesthetic or paracetamol (…) I’ve got to leave them in pain just that bit longer. I don’t think that’s to my full potential. (Participant 3)

Looking ahead, some participants spoke about how their current roles may expand within the ED in the future. For example, Participant 1 said:

I’ve been asked by the consultants whether I wouldn’t mind you know multi-tasking sort of thing as working in like in resus, majors and [paediatrics] as well. (Participant 1)

## Discussion

The changeover to working in an ED represented a significant period of role transition for the participants; the sense of being busier, changes in clinical responsibility and decision making were popular responses. Various authors recognise such transitions as being challenging, seldom straightforward and often stressful for the individual (Barnes, 2015; Cusson & Viggiano, 2002; MacLellan, Levett-Jones, & Higgins, 2015).

For one participant, the experience of moving to the ED had caused a sense of role regression, from a position of expertise within the ambulance service to that of a novice ED practitioner. Cusson and Viggiano (2002) warn that individuals must be prepared for the frustration caused by a sense of returning to the role of a student during role transition. Indeed, the work of Hamric and Taylor (1989) on the development of clinical nurse specialist roles identifies that following the initial excitement at the opportunity to apply new theory to practice, the nurse will experience frustration as they feel overwhelmed by the requirements of their new role and express the desire to return to their previous, more familiar position. These concepts substantiate the experiences described in this study and suggest that they form part of a natural process during role transition.

Many participants described the skills they believed transferred into their new roles from their pre-hospital paramedic practice, including those in physical assessment and diagnostic reasoning. This finding is congruent with those in a study by Perchie (2003) of paramedics working in a Canadian ED; skills relating to patient assessment and resuscitation transferred into the ED context, enabling paramedics to work effectively in those instances where such skills were necessary. The concept of transferrable skills is also found in a study of emergency nurse practitioners by Lloyd-Rees (2016), who described how their experience and knowledge as senior nurses also carried over to their new roles to the benefit of patient care.

All study participants noted how they had limited access to medicines in their new roles, agreeing that this presented the most significant challenge to their expanding practice. In the pre-hospital context, UK paramedics utilise specific exemptions within the Medicines Act 1968 to administer a range of medications autonomously and without the need for a prescription or PGD, as part of their normal scope of practice (Christie, 2015; Duffy, 2017). Unsurprisingly, many participants held the opinion that independent prescribing for paramedics would solve these issues. This view is supported by the experiences of nurses in a study by Jones, Edwards and While (2011), who described improved timeliness of patient care, improved patient satisfaction and reduced dependency on existing prescribers among the benefits of nurse prescribing acute care settings.

### Trustworthiness and limitations

The use of a well-established methodology such as phenomenology added to the rigour and sophistication of the research design; by adopting principles of descriptive phenomenology, the everyday conscious experiences of the paramedics could be described during data collection and subsequently presented. Furthermore, records of interview transcripts, coding frameworks and interview notes have been retained to comprise an audit trail relating to the data analysis process.

The small number of EDs included, with some participants working in the same department or for the same Trust (but at different EDs), may contribute to an element of commonality in the study findings. The study findings cannot reflect a true picture of the experiences of all paramedics working in EDs across the West Midlands region; however, the emphasis of this study was on the description of lived experiences rather than on producing generalisable findings.

### Recommendations and implications

Paramedics arriving in the ED setting will benefit from a more comprehensive induction programme that incorporates areas where paramedics have no experience, or less than their nursing colleagues, such as the functioning of the hospital and ED environment and familiarisation with IT systems, referral pathways and specialities. Since the completion of data collection, medicines legislation has been changed to allow advanced paramedics to become independent prescribers (College of Paramedics, 2018); the impetus is now on ED leaders to instigate this development. It will take time before the first paramedic prescribers emerge into clinical practice; therefore, it is important that existing arrangements for the supply and administration of medicines for non-prescribers are reviewed and that they reflect the contemporary mix of health professionals now working in the ED setting.

Future research incorporating a wider distribution of EDs with a more heterogeneous sample would broaden the understanding of this area of paramedic practice. Additionally, further research involving paramedics working in other areas, such as urgent care centres or out-of-hours services, would provide insight into whether they share or have different experiences of role transition and clinical practice.

## Conclusion

While role transition to the ED represents a turbulent period for paramedics, elements of paramedic practice transfer directly into new roles and contribute to effective practice. The paramedics in this study found that they were accepted and supported to work in the ED setting, and spoke positively of expanding their roles into other areas of the ED in the future. A significant barrier to current clinical practice emerges from a lack of access to medicines which impacts directly on the patient experience. The change in legislation to allow independent prescribing for advanced paramedics must be supported by ED managers, and interim improvements are required to extend existing PGDs to include paramedics; ultimately this will improve the quality and safety of care they are able to provide, as well as the patient experience.

## Acknowledgements

The author would like to thank Mr Peter Gregory (University of Wolverhampton) for his assistance as research supervisor for this project and Dr Julian Barratt (University of Wolverhampton) for his help with the technical editing of this manuscript.

## Conflict of interest

None declared.

## Ethics

Ethical approval for the study was granted by the authorising university’s ethics committee. Organisational approval was given by the relevant ED managers and university staff to approach potential participants, who were given information relating to the study before being invited to take part. Informed written consent was obtained from participants at the start of each interview.

## Funding

None.

## References

[bibr_1] Barnes H. (2015). Exploring the factors that influence nurse practitioner role transition. *Journal of Nurse Practitioners*, 11, 178–183. 10.1016/j.nurpra.2014.11.004 PMC4323084 25685113

[bibr_2] Birt L. Scott S. Cavers D. Campbell C. Walter F. (2016). Member checking: A tool to enhance trustworthiness or merely a nod to validation? *Qualitative Health Research*, 26, 1802–1811. 27340178 10.1177/1049732316654870

[bibr_3] Bryson C. (2016). How emergency department staff perceive acute nurse practitioners. *Emergency Nurse*, 23, 26–31. 26948226 10.7748/en.23.10.26.s23

[bibr_4] Choo E. Garro A. Ranney M. Meisel Z. Morrow Guthrie K. (2015). Qualitative research in emergency care part I: Research principles and common applications. *Academic Emergency Medicine*, 22, 1096–1102. 26284696 10.1111/acem.12736 PMC4545270

[bibr_5] Christie G. (2015). Independent non-medical prescribing for paramedics. *Nursing Standard*, 29, 36–39. 10.7748/ns.29.51.36.e9771 26509193

[bibr_6] College of Paramedics. (2015). *Paramedic – scope of practice policy*. Bridgwater, England: College of Paramedics.

[bibr_7] College of Paramedics. (2018). *Practice guidance for paramedic independent and supplementary prescribers*. Bridgwater, England: College of Paramedics.

[bibr_8] Converse M. (2012). Philosophy of phenomenology: How understanding aids research. *Nurse Researcher*, 20, 28–32. 10.7748/nr2012.09.20.1.28.c9305 23061271

[bibr_9] Creswell J. Poth C. (2018). *Qualitative inquiry and research design. Choosing among the five approaches* (4th ed.). Thousand Oaks, CA: SAGE Publications, Inc.

[bibr_10] Cusson R. Viggiano N. (2002). Transition to the neonatal nurse practitioner role: Making the change from the side to the head of the bed. *Neonatal Network*, 21, 21–28. 10.1891/0730-0832.21.2.21 11923997

[bibr_11] Duffy I. (2017). Exploratory study into the views of paramedics on paramedic prescribing. *Journal of Paramedic Practice*, 9, 296–301.

[bibr_12] Fisher J. (2006). Developing the A&E nurse practitioner role. *Emergency Nurse*, 13, 26–31. 16566315 10.7748/en2006.03.13.10.26.c1208

[bibr_13] Gallagher A. Vyvyan E. Juniper J. Snook V. Horsfield C. Collen A. Rutland S. (2016). Professionalism in paramedic practice: The views of paramedics and paramedic students. *British Paramedic Journal*, 1, 1–8.

[bibr_14] Griffin M. Melby V. (2006). Developing an advanced nurse practitioner service in emergency care: Attitudes of nurses and doctors. *Journal of Advanced Nursing*, 56, 292–301. 17042808 10.1111/j.1365-2648.2006.04025.x

[bibr_15] Hamric A. Taylor J. (1989). *The clinical nurse specialist in theory and practice* (2nd ed.). Philadelphia, PA: Wiley Blackwell.

[bibr_16] Health Education England. (2017). *Advanced clinical practice*. Retrieved from https://www.hee.nhs.uk/our-work/developing-our-workforce/advanced-clinical-practice/advanced-clinical-practice-definition.

[bibr_17] Hill H. McMeekin P. Price C. (2014). A systematic review of the activity and impact of emergency care practitioners in the NHS. *Emergency Medicine Journal*, 31, 853–860. 23851036 10.1136/emermed-2013-202660

[bibr_18] Jones K. Edwards M. While A. (2011). Nurse prescribing roles in acute care: An evaluative case study. *Journal of Advanced Nursing*, 67, 117–126. 21073502 10.1111/j.1365-2648.2010.05490.x

[bibr_19] Kodish S. Gittelsohn J. (2011). Systematic data analysis in qualitative health research: Building credible and clear findings. *Sight and Life*, 25, 56.

[bibr_20] Lloyd-Rees J. (2016). How emergency nurse practitioners view their role within the emergency department: A qualitative study. *International Emergency Nursing*, 24, 46–53. 26163111 10.1016/j.ienj.2015.06.002

[bibr_21] MacLellan L. Levett-Jones T. Higgins I. (2015). Nurse practitioner role transition: A concept analysis. *Journal of the American Association of Nurse Practitioners*, 27, 389–397. 25186979 10.1002/2327-6924.12165

[bibr_22] Mason S. O’Keeffe C. Coleman P. Edlin R. Nicholl J. (2007). Effectiveness of emergency care practitioners working within existing emergency service models of care. *Emergency Medicine Journal*, 24, 239–243. 17384374 10.1136/emj.2006.035782 PMC2658226

[bibr_23] Mason S. O’Keeffe C. Knowles E. Bradburn M. Campbell M. Coleman P. … Patterson M. (2012). A pragmatic quasi-experimental multi-site community intervention trial evaluating the impact of emergency care practitioners in different UK health settings on patient pathways (NEECaP trial). *Emergency Medicine Journal*, 29, 47–53. 22186262 10.1136/emj.2010.103572

[bibr_24] National Audit Office. (2011). *Transforming NHS ambulance services*. London: National Audit Office.

[bibr_25] NHS England. (2013). *High quality care for all, now and for future generations: Transforming urgent and emergency care services in England: Urgent and emergency care review end of phase 1 report*. Leeds, England: NHS England.

[bibr_26] Norris T. Melby V. (2006). The acute care nurse practitioner: Challenging existing boundaries of emergency nurses in the United Kingdom. *Journal of Clinical Nursing*, 15, 253–263. 16466474 10.1111/j.1365-2702.2006.01306.x

[bibr_27] Perchie G. (2003). *Paramedics in the emergency department* (Master’s dissertation). Retrieved from https://search.proquest.com/pqdt/results/2D1F897C67334FE4PQ/1?accountid=14685.

[bibr_28] Punch K. (2006). *Developing effective research proposals* (2nd ed.). London, England: SAGE Publications, Inc.

[bibr_29] Ranney M. Meisel Z. Choo E. Garro A. Sasson C. Morrow Guthrie K. (2015). Interview based qualitative research in emergency care part II: Data collection, analysis and results reporting. *Academic Emergency Medicine*, 22, 1103–1112. 26284572 10.1111/acem.12735 PMC4560670

[bibr_30] Ritchie J. Lewis J. McNaughton-Nicholls C. Ormston R. (eds.), (2014). *Qualitative research practice. A guide for social science students and researchers* (2nd ed.). London, England: SAGE Publications, Inc.

[bibr_31] Tavares W. Bowles R. Donelon B. (2016). Informing a Canadian paramedic profile: Framing concepts, roles and crosscutting themes. *BMC Health Services Research*, 16, 477. Advance online publication. doi: 10.1186/s12913-016-1739-1. 27605119 10.1186/s12913-016-1739-1 PMC5015210

[bibr_32] Tong A. Sainsbury P. Craig J. (2007). Consolidated criteria for reporting qualitative research (COREQ): A 32-item checklist for interviews and focus groups. *International Journal for Quality in Health Care*, 19, 349–357. 17872937 10.1093/intqhc/mzm042

[bibr_33] Tye C. Ross F. (2000). Blurring boundaries: Professional perspectives of the emergency nurse practitioner role in a major accident and emergency department. *Journal of Advanced Nursing*, 31, 1089–1096. 10840242 10.1046/j.1365-2648.2000.01380.x

